# Peptide-Based Nanoparticles for Systemic Extrahepatic Delivery of Therapeutic Nucleotides

**DOI:** 10.3390/ijms24119455

**Published:** 2023-05-29

**Authors:** Samuel A. Wickline, Kirk K. Hou, Hua Pan

**Affiliations:** 1 Division of Cardiology, Department of Medical Engineering, University of South Florida, Tampa, FL 33602, USA; 2 Department of Ophthalmology, Stein and Doheny Eye Institutes, University of California, Los Angeles, CA 90095, USA; 3 Division of Rheumatology, Department of Medicine, Washington University School of Medicine, St. Louis, MO 63110, USA; 4 Department of Pathology and Immunology, Washington University School of Medicine, St. Louis, MO 63110, USA; 5 Department of Biomedical Engineering, Washington University in St. Louis, St. Louis, MO 63130, USA

**Keywords:** nanoparticles, peptide polyplexes, RNA, siRNA, mRNA, nucleic acids, extrahepatic delivery, endosomal escape, peptide-based nanoparticles

## Abstract

Peptide-based nanoparticles (PBN) for nucleotide complexation and targeting of extrahepatic diseases are gaining recognition as potent pharmaceutical vehicles for fine-tuned control of protein production (up- and/or down-regulation) and for gene delivery. Herein, we review the principles and mechanisms underpinning self-assembled formation of PBN, cellular uptake, endosomal release, and delivery to extrahepatic disease sites after systemic administration. Selected examples of PBN that have demonstrated recent proof of concept in disease models in vivo are summarized to offer the reader a comparative view of the field and the possibilities for clinical application.

## 1. Introduction

Recent advances in the clinical application and regulatory approval of nucleotide therapeutics have launched a veritable avalanche of new research and commercial activity in the design of RNA species and delivery vectors for a myriad of diseases. Following initial excitement around the discovery of RNA interference [1], a decades-long effort to gain clinical traction for RNA as a pharmacological agent appeared stalled for numerous reasons, and the field was abandoned by all major pharmaceutical companies [2]. Although use of RNA species to inhibit the translation of messenger RNA (mRNA) advanced as a critical in vitro methodology for discovery of novel molecular targets, the quest to selectively deliver RNA to a chosen tissue or cell type in vivo was less successful. Nevertheless, a handful of companies including Alnylam, Ionis, Dicerna, Moderna, BioNtech, and others persisted and eventually developed selective delivery approaches for targeting hepatic diseases or as vaccines [3,4,5]. Concomitantly, novel modifications of RNA backbone structures were devised that both enhanced efficacy and reduced immune recognition [6,7,8,9,10,11].

To treat liver-based diseases with RNA, the principal breakthrough was the recognition that hepatocytes abundantly express a lectin, the asialoglycoprotein receptor (ASGPR), that could be targeted specifically with the use of novel ligand conjugates formed from *N*-Acetylgalactosamine (GalNAc) moieties, which are amino sugar derivatives of galactose [3,12]. Direct conjugation to the RNA itself (e.g., small interfering RNA—siRNA or antisense oligonucleotides—ASO) was shown to be sufficient for hepatocyte delivery and efficacy [13,14]. The first siRNA agent approved in 2018, which regulates the hepatic production of misfolded transthyretin that is responsible for transthyretin amyloidosis (ONPATTRO^®^, Alnylam; Cambridge, MA, USA), set the stage for the current resurgence and commercial progress in the field. Of course, a significant history of prior work on this targeting system preceded the commercial development of an effective delivery system for RNA, which is detailed in a recent review article [12].

Unfortunately, the problem of RNA delivery to cells other than hepatocytes still exists because the ASGPR-GalNAc system is not generalizable to other tissues. Some recent reports of conjugation of RNA or RNA delivery vectors to central nervous system (CNS)-targeting ligands (e.g., transferrin receptors [15], lipophilic hydrocarbon chains [16], or cholesterol [17]) suggest that inroads may be made in some cases, but these approaches are still early in design and evaluation. Other approaches to achieve extrahepatic delivery include direct local instillation, e.g., surgical implantation of depot delivery systems such as anti-KRAS siRNA in the “LODER “device for pancreatic cancer [18] or intrathecal injection of mRNA splice modifying ASOs for spinal muscular atrophy [9]. However, a robust paradigm for systemic administration of RNA that elicits selective homing to extrahepatic sites of interest is not yet available.

This delivery roadblock also affects adoption of systemic mRNA therapeutic strategies [19,20]. Because of its large size as compared to siRNA or ASO entities and its range of sizes that exceed even the ability of viral constructs to package more than 5000 nucleotides (nt), the prevailing opinion is that mRNA will need to be enveloped within a lipid nanoparticle (LNP) carrier to be effective for systemic applications [21]. Fortunately for the current generation of RNA vaccines, LNPs loaded with mRNA and containing ionizable lipid components are potent immunogens without the need for targeting [22], which represents another breakthrough that is driving the RNA pharmaceutics field. Nevertheless, directing LNPs safely to other extrahepatic targets while minimizing liver uptake is a formidable task as LNPs and other polymeric or composite particles are naturally cleared by the liver after systemic administration [23]. Moreover, interest in peptide-based approaches for larger RNA structures is gaining attention [24], as we review in selected examples below.

Other barriers exist along the path to effective extrahepatic delivery of RNA, but perhaps the major impediment is timely release of sufficient quantities of RNA from endosomal compartments to either engage the RISC complex for siRNA or ribosomes for mRNA. For example, when treating liver-based diseases such as transthyretin amyloidosis, the ASGPR is engaged by circulating GalNAc targeted siRNA and is taken up into early endosomal compartments to later be sorted into other unspecified storage compartments. Elegant studies by Alnylam scientists have described a process of low-grade and sustained endosomal release of depot siRNA reserves rather than rapid and complete burst release of siRNA [13]. The exact process responsible for the ultimate escape of GalNAc-siRNA from the endosome remains unclear, but it seems apparent that slow continuous release of siRNA allows for prolonged engagement of the RISC complex and months-long persistence of knockdown that are observed even after single doses. However, under other circumstances where cells are proliferating rapidly as in cancer or in prothrombotic atherosclerotic plaques that cause unstable angina, myocardial infarction, and stroke or in emerging infectious diseases, perhaps a more immediate and urgent effect for target knockdown would be prudent. Here, one might desire a formulation featuring accelerated endosomal egress of siRNA as single or even combination agents. Some potential approaches to this problem are addressed below.

In this review, we focus on RNA complexing approaches that utilize generalizable peptide-based nanoparticle (PBN) structures to facilitate extrahepatic delivery after systemic administration. In general, polyplex structures self-assemble when polycations interact with negatively charged nucleic acids to form polyion complexes. The rationale for peptide-based self-assembling nanostructures resides in the great flexibility for controlling important features such as charge, amphiphilicity, pH sensitivity, and reactivity that are described below, which are specific to selected promising formulations reviewed herein. Moreover, the ability to modify, produce, and evaluate these features easily, rapidly, and cheaply for improving performance presents distinct advantages.

We also examine how such constructs might enhance endosomal escape while protecting RNA in circulation. These types of peptides generally might be considered as members of the broad class of “cell penetrating peptides “ (CPP) or “antimicrobial peptides “ (AMP), but there are some distinguishing features that set them apart. Numerous reports and review articles are available that describe the classification, heterogeneity, and uses of CPP and AMP for reference [25,26,27,28,29] in addition to their use in modifying other nanostructures by direct conjugation [30,31,32]. For the most part, these are moderate size (<50 a.a.), cationic, amphipathic peptides that were recognized as natural components of biotoxins (e.g., melittin, in honeybee venom), [26,33,34], as host defense peptides [25,27], or as natural vectors promoting cell membrane penetration of various cargos including viruses (e.g., TAT: cell-penetrating peptide with sequence of RKKRRQRRRR, in HIV) [35,36,37,38]. Humans also harbor natural membrane-disrupting peptides for immune defense such as the gasdermin-D that participates in pyroptosis by oligomerization and membrane pore formation after proteolytic cleavage and activation [39,40,41]. For our purposes here we focus more on the membrane-inserting or membrane-disrupting capabilities of cationic amphipathic peptides, which does not require that they perform as transmembrane porters of RNA per se but rather promote endosomal membrane destabilization and RNA escape following uptake by active endocytotic mechanisms. Although the cationic amphipathic pore-forming peptide melittin has not succeeded as a viable PBN formulation, in this review we shall lean on it and its safer modified analogues for mechanistic insights as a classic CPP/AMP because of the extensive library of experimental and modeling work regarding its physical interactions with lipid membranes and other molecules [42].

We also review those PBN that feature self-assembly of highly stable nanostructures resulting from combined electrostatic and hydrophobic interactions between amphiphilic/cationic peptides and anionic nucleotides. Alternative strategies, prospects, and challenges for covalent conjugation of CPP to RNA moieties are covered in other reviews [43], but the flexibility to alter or multiplex cargos through a self-assembling mixing process presents advantages for rapid product design, formulation, and testing. We will deal primarily with siRNA and mRNA or plasmid DNA (pDNA) as representative small and large nucleotide structures that can form PBN. Because some formulations have existed for decades and many have never advanced to in vivo testing, we will concentrate on exemplifying PBN systems that have reported in vivo proof of concept using systemic administration for multiple disease applications in recent years. For readers interested in nanoparticle vaccines and formulations with CPP for transcutaneous or intradermal administration, recent reviews are available [22,44].

## 2. PBN Self Assembly

Most peptides that have been reported to self-assemble into stable nanoparticles are cationic and amphiphilic (See Table 1). Primarily amphiphilic peptide constructs may comprise separated sequences of hydrophilic and hydrophobic amino acids. Alternatively, modification by addition of hydrophobic fatty acid chains or other hydrophobic entities (e.g., cholesterol, myristoyl, stearyl, etc.) to synthetic polybasic amino acid sequences yields primary amphipathic vectors. Branched peptide structures are more complicated as they might contain more complicated spatial domains of hydrophobic and hydrophilic elements. We describe in vivo working examples of both below.

**Figure 1:**
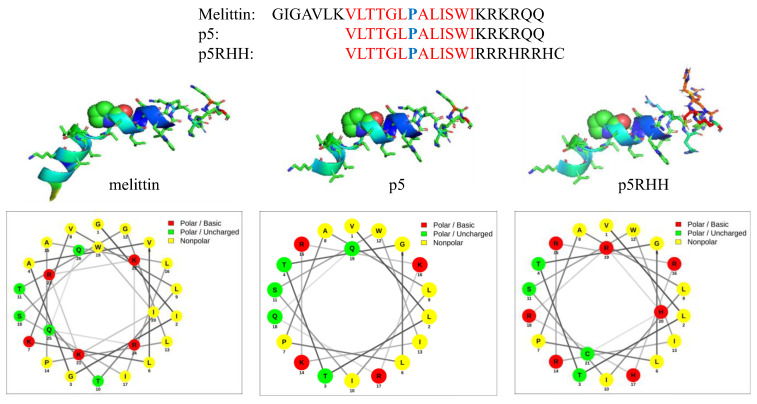
Amphiphilic Peptide Examples: Melittin, p5, and p5RHH sequences. Both the primary and secondary amphiphilicity is exhibited by melittin and its modified versions, p5 and p5RHH. Peptide helical wheels were generated by using a web application [53].

Amphiphilicity also can be induced dynamically when peptides undergo alterations in secondary structure to assume alpha helical or other forms. In “secondary amphiphilicity “, a separated alignment of hydrophobic and hydrophilic residues segregates on opposite “faces “ of a helical structure in response to changes in pH, local environment, or enthalpic/entropic forces. An example of both forms of amphiphilicity is found in the natural peptide melittin, which is a 26 amino acid cationic component of bee venom that manifests membrane inserting and pore forming functions that are useful for therapeutic applications [33,42,45,46,47]. In the native sequence, the hydrophobic N-terminal residues and the hydrophilic C-terminal residues are separated in random coil configuration. However, upon assuming alpha helical structure in lipidic membranes, prominent facial separations on opposite turns of the helix also come into play (Figure 1). Early attempts to incorporate melittin into siRNA polyplex formulations by Arrowhead Pharmaceuticals utilized a metabolizable melittin masking moiety to reduce toxicity until unmasked in an endosome at acidic pH [48], but this approach ultimately failed for safety reasons in clinical trials, and it was abandoned. Interestingly, around that time the Wickline group working with melittin as an anti-cancer agent [45,46,49] already had designed a modified version of melittin for stable membrane insertion and molecular cargo delivery that improved safety by nearly three orders of magnitude by truncating seven N-terminal hydrophobic residues (“p5“ in Figure 1). Subsequent modification of C-terminal basic residues preserved both primary and secondary amphiphilicity and enabled PBN formation and transfection with siRNA in (“p5RHH“ in Figure 1) [50,51,52]. This new 21 amino acid, cationic peptide sequence condensed nucleotides into a pH-sensitive structure that disassembles only in endosomes to release the p5RHH peptide component, which then permeabilizes the endosomal membrane as acidification progresses. This formulation now has been used successfully by many collaborating academic groups for systemic delivery of siRNA and mRNA extrahepatically across a broad array of pathologies as described below.

Amphipathic PBN peptide features are important not only in interactions with lipid membranes in the manner of cell penetrating peptides but also in noncovalent interactions with nucleotides [54]. Attractive Coulombic interactions between cationic peptides and anionic nucleotides initiate associations, which then are consolidated by hydrogen bonding, hydrophobic, and other forces available for noncovalent peptide interactions [55,56,57,58]. An incubation period of 30–60 min appears typical in most reported batch process formulations to complete the process of nanostructure self-assembly. The exact ratios of peptide to nucleotide, which are expressed either as the charge ratio of peptide nitrogen to nucleotide phosphate (N:P) or in molar ratios, are specified to achieve a desired particle size, charge, and stability that are critical for transfection. Depending on the peptide length, charge, amphiphilicity, exact amino acid composition, salt concentration, and other features, these ratios can vary considerably.

As an instructive example of PBN self-assembly, the association of the cationic amphipathic CADY peptide with siRNA is considered [59]. CADY (Ac-GLWRALWRLLRSLWRLLWKA-cysteamide) is a 20-residue cationic peptide that assumes an alpha helical secondary structure in lipidic environments. Upon association with siRNA in 20- or 40-1 ratios, small spherical units (20 nm) emerge that then accrete into a ~156 nm superstructure (charge: ~+50 mv) that is further stabilized by an additional surrounding CADY peptide corona, which is referred to as a “raspberry“ [60]. Model-based calculations suggest that 10–13 peptides interact electrostatically with each siRNA. The tryptophans appear important to this interaction as they partially quench and blue shift their emission spectra upon close interaction with siRNA. CADY exhibits high affinity for siRNA (Dissociation Constant (K_d_): ~15 nmol/L), and the compacted polyplex protects RNA against nucleases in circulation.

Recent work by Ratnayake et al., has explored in depth the docking interactions between various cell penetrating peptides and siRNA in the formation of PBN structures [61]. By calculating binding energies for cationic, amphipathic, and hydrophobic peptides interacting Coulombically with model siRNA molecules, a general dynamic was observed: initial binding of peptides onto siRNA was energetically favorable, while the magnitude of the binding scores generally decreased as more peptides complexed with the siRNA. Serial stages of peptide-siRNA binding were elucidated:

(1) Electrostatic and peptide shape factors initiate binding of cell penetrating peptides into the major groove of siRNA, which is the locus for the maximal salt bridges, but only 2–3 peptides can bind to the major groove due to steric constraints.
(2) Subsequent binding of peptides occurs at the minor groove with some arranged perpendicular to the siRNA. Peptides with greater + charge favored minor groove binding at this point.
(3) As the negative surface charge on the siRNA becomes progressively screened, further aggregation of peptides coating the complex can occur by emerging hydrophobic peptide–peptide interactions, and the size of the overall siRNA-peptide complex can increase.

Additional observations are worth noting from the report. First, the predicted secondary structure of the peptide had minor bearing on the RNA binding energy scores in that alpha helices, beta sheets, and random coils all had similar binding energies. However, alpha helices such as CADY manifested higher binding efficiencies throughout the range of peptide number associations up to 30:1. Second, hydrophobic effects between bound and free peptides create more favorable accumulation of additional peptide into PBN complexes for amphipathic versus simple cationic peptides.

The authors opined that the shape of the peptide complex is an important factor in that helical “tilts“ would fit better into the RNA major groove to stabilize the interactions by hydrogen bonding (to 2′-OH groups and exposed nitrogen base pairs in grooves) and by hydrophobic forces. In this regard, the potential role of proline residues to form a more conforming 30-degree kink or tilt in helical peptide structures is discussed below. Additionally, the affinity of peptides for nucleotides can vary markedly with salt concentration, which implies that electrostatic interactions and PBN stability will be affected by the local concentration of charge neutralizing positive ions or molecules [62]. This behavior accords with the calculated reduction in binding energies observed by Ratnayake et al. as amphipathic cationic peptides accumulate on siRNA backbones [61]. The local charge effect also has implications for pH responsiveness of histidine containing peptides as described below.

The above remarks predominantly deal with oligonucleotide formulations of <30 nt, but not with larger structures such as mRNA or DNA. Moreover, this modeling illustrates the restricted interactions of one nucleotide to a collection of peptides but does not envision more complex polyplex formations. However, hydrophobic and hydrogen bonding forces among nucleotide-accreted peptides are likely to aggregate these individual peptide-RNA units together over time as the polyplex “matures“.

**Figure 2:**
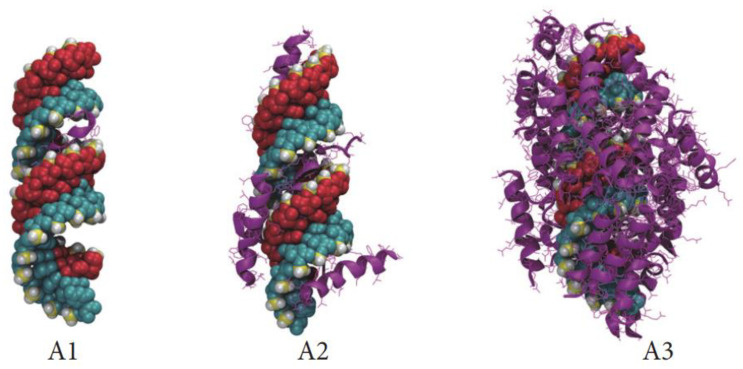
Predicted structures of CPP and anti-GAPDH siRNA in A-form complex from docking calculations. Pictures show the CADY peptide representing the amphipathic class of CPPs. The peptide to siRNA ratios are 1:1 (**A1**), 5:1 (**A2**), and 30:1 (**A3**), respectively. From Rathnayake et al. [10].

Chou et al. from the Mixson group examined the thermodynamics of PBN formation with calorimetry to identify three discrete stages of peptide–siRNA interaction [63]. The first stage, exothermic and enthalpy driven, accords with initial electrostatic binding events between naked siRNA to glyceraldehyde-3-phosphate dehydrogenase (GAPDH) and CADY peptides with peptides filling in major and minor grooves as elucidated above by Rathnayake (Figure 2A1,A2). An exothermic transition occurs shortly thereafter, likely representing peptide-peptide interactions at the site of a single siRNA as the siRNA charge becomes screened by continuing peptide accretion (as per Figure 2A3). The final transition was endothermic and entropy driven, concomitant with the emergence of polyplexes identified by dynamic light scattering measurements. At this point, unitary peptide-siRNA assemblies are aggregating, similar to the “raspberry “ stage described by Deshayes above.

Several examples of plasmid DNA PBN are described below, but mRNA remains poorly exploited as a PBN formulation. At this point however, there is little solid evidence supporting the promise of lipid nanoparticle formulations for mRNA delivery beyond their clear utility as vaccine modules. In fact, one might argue that the ability to incorporate only a few (~2–3) mRNA into single lipid nanoparticles [64] would militate against their efficacy in vivo. However, there are several commercial lipid nanoparticle mRNA formulations in Phase I and II clinical trials for cancer [65], so an answer on efficacy should be forthcoming. For PBN formulations, recent work with the amphipathic cationic peptide p5RHH has shown promise for ready formulation of mRNA that demonstrates potent extrahepatic delivery and efficacy after systemic administration as described below [66,67]. Other PBN systems for delivery of plasmid DNA are described below.

## 3. Functional Aspects of Key Amino Acids in PBN Formation

### 3.1. Arginine and Lysine

Arginine (**R**) and/or Lysine (**K**) are charged basic residues commonly found in amphipathic cell-penetrating and membrane-inserting peptides that establish the necessary conditions for the initial electrostatic interactions with negatively charged nucleotides. Positive residues also initiate interactions with negatively charged cell membranes to induce changes in peptide secondary structure, membrane insertion, nanoparticle-membrane fusion, or uptake by active and passive endocytotic mechanisms. Despite being a potent peptide toxin in free form, melittin can be stably loaded into the surfactant coating of hydrophobic/lipophobic perfluorocarbon nanoparticles for safe cytoplasmic delivery [45,46,49]. However, in our hands it was not able to be formulated into a sufficiently transfective nanoparticle for nucleotide delivery (K.K. Hou: unpublished data).

Many versions of poly-lysines and poly-arginines have been deployed in synthetic amphipathic constructs and other derivatives of natural peptides (e.g., TAT, transportan, etc.) for covalent and noncovalent nucleotide delivery as described in other reviews [28,68,69,70]. Some have suggested that **R** residues may be preferred based on their prevalence in natural cell penetrating peptides, their stronger hydrogen bonding capacity, and their ability to effect membrane translocation and internalization even at 4 degrees [71,72,73]. In any event, consideration of toxicity for highly positively charged nanoparticles is paramount for systemic delivery to avoid inflammatory consequences, off target deposition, or rapid clearance that might accompany other delivery systems [74].

**R** in fact is notable for being the most hydrophilic of basic amino acids [75]. It is polar and positively charged at neutral pH, and its guanidinium side chain can engage in up to six hydrogen bonds. In peptides that form alpha helices with facial amphiphilicity in lipid membrane environments, **Rs** can initiate pore formation for peptides such as melittin through initial electrostatic interactions with lipid head groups at membrane surfaces [27,33]. However, hydrogen bonding to lipid phosphate oxygens and esters as acceptors to arginine guanidium donors might facilitate lipid bilayer deformation in the process. Together with the proline “kink“ in the middle of the melittin structure that permits some flexibility for the now helical facially amphiphilic peptide to situate in lipid bilayers (see below), lipid head groups can be pulled into the interior of the bilayer to form toroidal, U-shaped, or hourglass type pores as described below by Tuerkova et al. [76].

### 3.2. Histidine

Histidine (**H**) plays an important role in the pH responsivity of PBNs, hydrogen bonding, disassembly, and endosomal escape due primarily to its aromatic side chain imidazole group. Histidine is capable of cation–π, π–π, salt-bridge, and other noncovalent interactions that stabilize polyplexes by the aromatic and hydrogen bonds that the imidazole group can establish. Midoux, Monsigny, and colleagues first designed a histidine containing peptide, H5WYG (GLFHAIAHFIHGGWHGLIHGWYG), that enhanced transfection of a polylysine-DNA complex without being associated with the polyplex itself [77]. Upon acidification, **Hs** were protonated; the peptide became positively charged, and conformational changes were noted in the peptide secondary structure. Permeabilization of cells in vitro was observed at acid pH but not under neutral conditions. A sharp concentration dependence for membrane disruption also was observed.

Bechinger and colleagues have examined the influence of pH on membrane insertional dynamics for peptides with incorporated **H** with the use of cationic amphipathic peptides of the LAH4 family [78,79]. The core of the LAH4 peptide is composed of leucine (**L**), alanine (**A**), and histidine (**H**) in repeats (26-mer: KKALLALAL**HH**LA**H**LAL**H**LALALKKA), where alpha helices are formed in membrane environments, and the **H** acid dissociation constant (pKa) values range from 5.4 to 6.0 to establish pH sensitivity. The lysine (**K**) side chains at either end condense DNA into a ~100 nm PBN where ~1 peptide complexes with every 2 bases in a 7600 bp plasmid DNA (or, N:P = 3800:1) [80]. In vitro, these complexes are taken up by endocytosis, and upon acidification and histidine protonation, the peptide charge increases to +9 from +5, and the DNA dissociates from the peptide. The authors suggest that the “overcharged “liberated peptides now in sufficient concentration interact with phospholipid head groups and ultimately permeabilize the endosomal membrane by a detergent surfactant action that allows DNA egress [81,82]. Similar results and mechanisms were observed for siRNA delivery in vitro [83].

**Figure 3:**
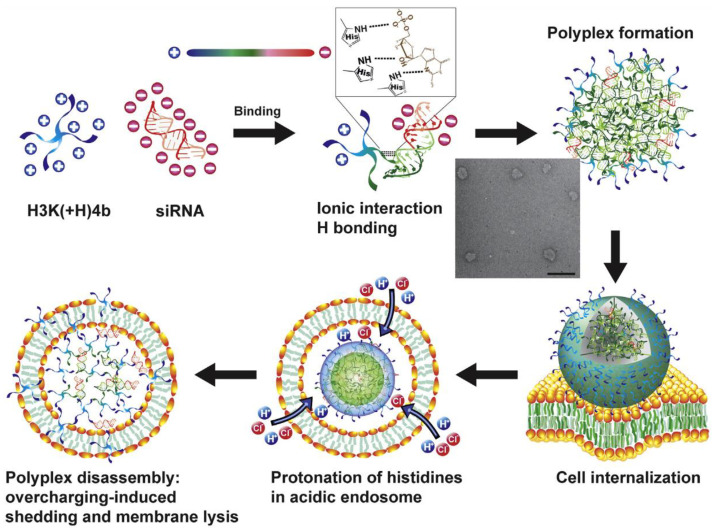
Branched HK-rich peptides binding siRNA. pH triggered endosomal escape by histidine protonation and charge accumulation. The colors blue, green, and red represent positive, neutral, and negative electrostatic potential, respectively. Hydrogen bonds are shown as dashed lines. Scale bar in the TEM FIG., 500 nm. From Chou et al. [63].

Other mechanistic observations on the hydrogen bonding properties of **H** in PBN complexes have been described by Mixson and colleagues. Chou et al. reported enhanced stability of linear histidine-lysine (“**HK**“) rich polyplexes forming nonionic hydrogen bonds with siRNA via the imidazole group nitrogens that exhibited improved transfection capacity (Figure 3) [63]. The initial interaction between **H** imidazole and siRNA was exothermic and energetically favorable. The authors opined that unpacking of the polyplexes was likely due to “overcharging “consistent with Bechinger’s explanation above and the modeling studies of Rathnayake (above) indicating reduced binding energies with increasing positive charge. Accordingly, protonation would promote charge–charge repulsion of the polyplex constituents, resulting in the break-up of the polyplex.

Hou et al., around the same time had postulated a similar behavior for the cationic amphipathic p5RHH system that incorporates only two histidines [51,52]. They argued that the process of polyplex disassembly was unlikely to involve the putative “proton sponge “mechanism (see below) and more likely to entail direct peptide-membrane surfactant interactions resulting in permeabilization and siRNA release, consistent with the Bechinger and Mixson explanations. This minimized **H** load perhaps might establish a lower limit for certain cationic amphipathic peptides as a necessary condition for endosomal pH-based polyplex disassembly, which is critical to the release of complexed RNA and biological efficacy. In other peptide structures with higher **H** loads, proton buffering and the osmotic sponge effect perhaps could dominate.

### 3.3. Tryptophan

Tryptophan (**W**) is felt by many authors to play an important role in cellular uptake through their noncovalent interactions with membrane lipids and cholesterol and in stabilizing nucleotide interactions through pi-bonding of the aromatic indole groups such as in WRAP peptides (see below [84]). **W** is also responsible for a portion of melittin’s membrane permeating capacity, depending on where additional **Ws** are added to the sequence [85]. Once melittin has adsorbed to membrane surfaces, it rapidly and strongly inserts into lipid monolayers in predominantly helical conformations with **W** deeply buried in the hydrophobic core as shown by Lee et al. in molecular dynamic simulations [86]. Ziegler and Seelig [87] and Bechara et al. [88] showed that **W** in certain cationic CPP strongly promotes cell internalization via initial electrostatic interactions with cell surface glycosaminoglycans, which can then direct them to clustered membrane domains for uptake.

In an in vitro study of the membrane binding of synthetic **R/W** nonapeptides, Walrant et al. determined that these cationic peptides bind to model lipids (POPG: palmitoyl-oleoyl-phosphoglycerol) and heparin (a glycosaminoglycan mimic) with increasing binding enthalpy as the peptide content of **W** increases [89]. In lipid vesicles, the peptides exerted disorganizing forces that reflected the extent of **W** substitution for **K**. Moreover, more **W** (3–4) in the peptide was associated with increased cellular membrane permeation and cytotoxicity as ion pair-pi bonding interactions prevailed. Secondary (facial) amphiphilicity was not required for these effects.

Jobin et al. examined the effects of replacing **W** in a synthetic **R/W** nonapeptide on internalization and cytotoxicity [90]. Both the number of **W** residues and their positioning in the helix were important for internalization, which increased up to three **W** residues. All studied peptides were partitioned into lipid membranes just below the lipid headgroups, but alterations in secondary structure did not impact internalization. The viability of Cho-K1 cells in culture was reduced somewhat over incubation times of 6–24 h, although liposomal integrity was not affected by pore formation.

### 3.4. Proline

Proline **(P)** residues in alpha helices tend to break or kink a helix formation to force a bend of about 30° in the helix’s axis to establish a helix-hinge-helix structure. Because P has no amide hydrogen, it cannot donate an amide hydrogen bond, while its sidechain also sterically hinders the helix backbone. Previous work by Lee et al. has shown that the introduction of **P** into a short amphipathic negative peptide induces a “kink “in the alpha helical structure [91]. This substitution facilitates micropore formation (<2 nm) and membrane translocation of the peptide rather than more typical membrane insertion. In this case, antimicrobial activity was enhanced through membrane penetration and subsequent cytoplasmic and nuclear interactions, although red cell hemolysis was not affected, consistent with formation of a small and transient pore.

Interestingly, melittin also features a **P** hinge in the middle of its 26 a.a cationic amphipathic sequence that separates hydrophobic from hydrophilic residues [26,27,49,92]. Pan et al. from our group mutated the melittin sequence as P^14^ > A^14^, which reduced its cell lytic activity in vitro to <10% of that for native melittin based on lethal concentration at 50% (LC50) values for cell viability, indicating the significance of proline in this modified structure (“p5 “) for membrane disruption [50]. Subsequent mutational iterations on melittin by Pan and Hou resulted in a sequence that was <50% homologous with melittin yet retained the proline to function as a cationic, amphipathic peptide capable of forming a PBN that promotes extensive and rapid endosomal escape [36,50,51,52,69]. An overall increase in safety margin of >300-fold in terms of LC50 was observed for the final proline-containing iteration, named as “p5RHH“ (see below).

**Figure 4:**
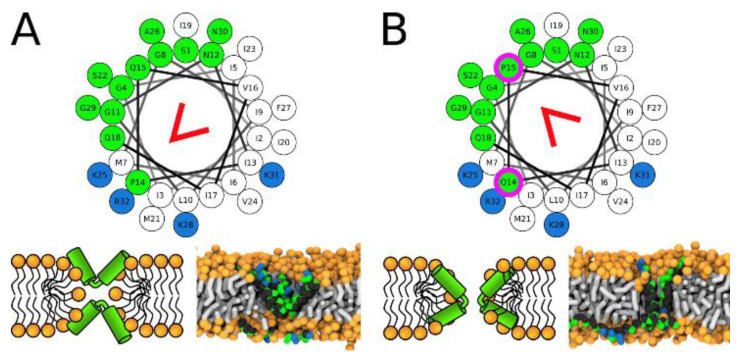
Proline hinge position affects pore structure. Here, Figure 8 in Tuerkova et al., illustrates the effects of altering the proline position in the peptide Candidalysin, which is a cytolytic toxin produced by Candida albicans. Simulations of CandKR peptide variants forming pores in 1-palmitoyl-2-oleoyl-*sn*-glycero-3-phosphocholine membranes are shown: (**A**) Wild Type and (**B**) P14Q/Q15P. In (**A**) the Wild Type forms a U-shape pore, whereas in (**B**) proline repositioning yields an Hourglass pore. Red wedge shows position of the proline kink. The positions of substituted residues are highlighted with magenta circles. Color coding of snapshots: hydrophobic residues = dark gray, hydrophilic residues = green, positively charged residues = blue, lipid phosphate group = orange, and lipid tail = light gray. From Teurkova et al. [76].

Recent structural investigations of the role of proline and/or glycine in membrane disruption by Tuerkova et al., indicate that peptide kinks provide flexibility for enhanced toroidal pore formation but can be destabilizing for barrel stave pores. Moreover, the exact pore configuration depends in part on where the kink is located (Figure 4) [76]. The exact position of the peptide kink affects these generalizations. The authors modeled several peptide structures to determine that most pore forming peptides formed toroidal type pores that were favored by the peptide kinks. Accordingly, they concluded that most pore forming peptides would benefit from insertion of a hinge region to improve membrane destabilization features if that was the goal.

## 4. Extrahepatic Delivery

Although the majority of polyplex nanoparticle structures and lipid nanoparticle-based carriers find their way to the liver for clearance, the direct conjugation of GalNAc moieties to the carriers or to siRNA cargos themselves appears to have adequately addressed the problem of specific hepatocyte uptake. However, extrahepatic delivery with systemic administration remains a significant roadblock to clinical translation of all nucleotide delivery approaches, especially for mRNA. Molecular targeting with selective ligands covalently coupled to delivery vehicles is a standard approach using conventional chemistries [93], yet problematic issues persist. For example, most targetable cellular receptors are ubiquitous, and even if they are overexpressed in pathological tissues such as cancer (e.g., CD44 for hyaluronic acid), they are abundantly expressed elsewhere in normal tissues in far greater quantities that might outcompete specific binding to smaller targets of interest. Moreover, as most PBN are impermeable to vasculature with physiological barrier function, molecular targeting to cell surface epitopes beyond the vasculature typically will depend initially on the EPR effect (**E**nhanced **P**ermeability and **R**etention) before encountering a desired cellular target [94,95,96]. Some strategies have been reported to actively facilitate vascular permeation such as the CendR approach (see below) [97], but it is not clear how robust, generalizable, and efficacious these will be as a universal modality for targeting diverse diseases.

Abundant review articles are available that describe the potential molecular targets that are reported to address cancer and other cells and organs of interest [98,99,100]. However, even if targeting enriches selective cellular uptake, most of the nanostructures still will be cleared by Kupffer cells and liver scavenger endothelial cells [101,102] of the reticuloendothelial system (RES). These scavenger cells recognize foreign bodies through several receptors including Toll-like receptors, scavenger receptors, Fc receptors, and so forth. As an unavoidable consequence of protein corona accretion on nanoparticle surfaces in vivo, opsonins (e.g., immunoglobulins, complement, and lipoproteins) accumulate and engage clearance mechanisms. One resort is to couple polyethylene glycol (PEG) to the surface to avoid RES sequestration and clearance [103]. However, PEG usage may be limited by toxicity associated with antigenic reactions to preformed PEG antibodies in patients with prior exposure or by accelerated blood clearance after repeat administration [104].

Besides the protein corona, among the more important physical features that dictate nanoparticle uptake are its native charge and shape, along with the serum derived protein, glycoprotein, and lipid corona components that immediately cover the nanoparticle after it is administered intravenously [23]. Charge and shape considerations have been described before [105], but for PBN, almost all are roughly spherical composites with sizes in the range of 40–200 nm. Outside of this range either kidney (smaller) or liver (larger) clearance likely will dominate. In terms of charge, exogenously formed PBN spans a gamut from highly negative to highly positive surface potentials. Moreover, these charges are likely to change after corona coating occurs, as might happen when negatively charged albumin invests and screens a positively charged PBN such as p5RHH (see below). For example, an early study on model peptide-based micelles by Xiao et al. found that highly positive (+37 mv) or negatively (–27 mv) charged spherical nanoparticles (15–20 nm) were taken up avidly by macrophage cell lines in vitro and liver Kupffer cells in vivo [106]. However, for slightly negative charged micelles (<10 mv), liver uptake was minimized, and tumor uptake was enhanced. Opsonization (i.e., corona formation) with mouse serum increased uptake of negatively charged micelles but decreased uptake of positive micelles.

Regardless of specific nanoparticle composition or molecular targeting, the EPR effect is likely to be responsible for the initial distribution of PBN to pathological tissues due to the permeability effects of inflammation on vascular barrier function in most pathologies [107,108,109]. Of course, one caveat is that some pathologies may not exhibit prominent EPR effects, such as genetic neurological diseases that retain a relatively intact blood–brain barrier. However, in inflammatory pathologies such as cancer, immature and leaky angiogenic neovasculature has been identified as the quintessential condition for the EPR effect. In other pathologies such as atherosclerosis, advanced lipid and macrophage-laden plaques are highly inflammatory, and local vascular barrier function is disrupted [110,111]. Glycocalyx deterioration [112], relaxation of adherens and/or tight junctions [108], and even late-stage endothelial death and sloughing [113] permit ready penetration of 200–250 nm or larger particles for diagnostic and therapeutic purposes [110,114,115,116,117].

Despite potential opportunities for EPR-based delivery, local extrahepatic deposition depends in part on the specifics of nanoparticle multicompartmental pharmacokinetics that avoid early sequestration by the RES to permit sufficient deposition in non-hepatic sites primed for EPR. Interest in manipulation of nanoparticle corona formation has emerged as one approach to channel extrahepatic compartmentalization [118,119]. Baimanov et al. recently have elucidated the in-situ dynamics of multilayered corona formation on small copper sulphate nanoparticles [120]. They identified an initial “hard corona “that avidly binds serum proteins to the nanoparticle surface, which is followed by envelopment of a lower affinity bound “soft corona “based on protein–protein interactions. These layers may remodel over time as continuous adsorption, exchange, and rearrangement of corona composition. Albumin was a major component of both hard and soft coronas, although myriad other immunoglobulin, complement, and other unidentified proteins were involved in a dynamic remodeling process over 30 min. The overall goal of such efforts is to elucidate specific surface features of nanoparticles that might promote selective accumulation of desirable corona components during in vivo circulation. However, given the likely variability of serum protein, lipid, and immunoglobulin composition from patient to patient, selective post hoc tailoring of the corona presents challenges to achieving specificity for molecular targeting.

An alternative approach is to pre-coat nanoparticles with various components that can be specified in advance in an effort to improve selective compartmental trafficking [118,121,122,123]. Dysopsonins such as albumin or various apolipoproteins can be preloaded onto nanoparticle surfaces prior to injection to establish a hard corona that enhances circulation times, reduces toxicity, and avoids RES clearance [124,125,126,127,128]. Hou et al., reported that PBN formed from p5RHH peptides and siRNA that were pretreated with albumin to form a hard corona enhanced transfection in vitro, while stabilizing PBN size and reducing charge to mildly negative (–5 to –7 mv) as suggested earlier by Xiao et al. [106] for enhanced extrahepatic delivery [51,52]. Subsequent studies in vivo with systemically administered p5RHH-RNA PBN confirmed avoidance of liver uptake and a shift to kidney clearance for the p5RHH system [66,114,129,130].

## 5. Endosomal Escape

The role of cell penetrating or membrane inserting peptides in breaching cellular and subcellular organelle membranes has been the subject of extensive biophysical investigation over the last 30 years [131]. Although recent reviews have summarized many of the putative mechanisms based on experimental and model-based approaches, precise allocation of sequential steps is challenged by necessarily simplistic assumptions when using homogenous lipidic vesicles or supported lipid bilayers in experimental and modeling approaches. However, relevant generalizations have emerged from such studies.

Most researchers now believe that endosomal uptake is the primary mechanism for cell entry of PBN and other nanoparticle carrier systems. This can take place in a variety of ways from macro-/micro-pinocytosis, clatherin- or caveolar-mediated uptake, and direct membrane fusion, among others [132]. Subsequent sorting of nanostructures in early and late endosomes has been covered elsewhere [133,134], but several sequential steps must be accomplished for PBN to effect endosomal release of the nucleotide.

First, the endocytosed nanostructure must survive a progressively acidifying endolysosomal environment. How much damage might occur to a single nanostructure over time as it transits endolysosomal pathways is difficult to define experimentally, but the general assumption is that the process of condensation of peptide and nucleotide protects the complex against nucleases and proteases, as has been reported for many of the designs when exposed to serum or concentrated nucleases (e.g., p5RHH [51,52,66]). Furthermore, a now standard array of RNA chemical modifications affords additional longevity to the nucleotide cargo [6,7,8,9,10]. In most cases, the strategy for PBN design is to create a complex that employs the peptide excipient itself as the endosomal permeabilizing entity in an effort to promote rapid escape. It is interesting to note that rapid and extensive early escape is the converse of what happens with ionizable lipid nanoparticle nucleotide complexes that are retained in endosomes, which appears to favor slow release over time for continuous cytoplasmic shuttling of small amounts of nucleotides [13].

**Figure 5:**
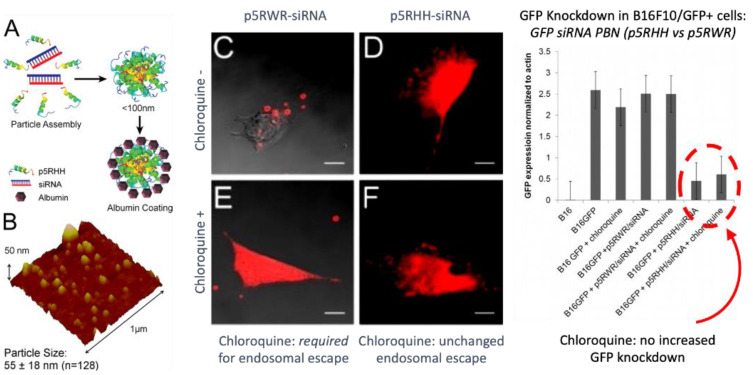
p5RHH formation, endosomal escape, and transfection. (**Left**)*:* (**A**) Particle assembly with a selected N:P ratio forms a mature polyplex within 40 min followed by coating with an albumin corona, as shown in the atomic microscopy image in (**B**). (**Middle**)*:* The ineffective p5RWR peptide forms siRNA nanoparticles but cannot escape from endosomes unless treated with chloroquine (note punctate appearance in (**C**) emerging into cytoplasm as diffuse distribution in (**D**)). Chloroquine releases sequestered endosomal siRNA contents for the ineffective PBN nanostructures (**E**), but no additional release is noted for the effective p5RHH nanostructures (**F**) because the p5RHH peptide-siRNA complex naturally escapes endosomes. (Confocal microscopy scale bars: 10 mm). (**Right**)*:* Anti-GFP siRNA knockdown in GFP-expressing melanoma cells is extensive with p5RHH polyplexes but not augmented by chloroquine, indicating that siRNA release is complete. However, p5RWR polyplexes cannot transfect and knockdown even with chloroquine. The addition of tight binding arginines and tryptophan to p5RWR, together with the lack of histidines that blocks pH-dependent peptide protonation, prevents particle disassembly and compromises efficacy. Adapted from Hou et al. [51,52].

Second, the complex must disassemble at some point to release free nucleotide and peptide excipient. This step presupposes that the overall binding energies (electrostatic, hydrophobic, hydrogen bonding, etc.) are not so strong as to prevent disassembly in the first place; otherwise, the nucleotide would never be active. This potential hurdle has been illustrated by Hou et al. for modified versions of their PBN peptide (Figure 5, left panel, p5RHH (VLTTGLPALISWIRRR*H*RR*H*C), by removing the two histidines and adding five arginines and a tryptophan to create the peptide “p5RWR “(VLTTGLPALISWIKRKRQQRWRRRR), which is the truncated (N-terminal 7 a.a.) version of melittin with “RWRRR “added to the C-terminus. This peptide forms an siRNA PBN that cannot escape the endosome on its own but only does so upon addition of chloroquine to release it into the cytoplasm (Figure 5, middle panel). However, even after the p5RWR PBN diffuses into the cytoplasm, the siRNA remains bound to the PBN polyplex and is not transfective (Figure 5, right panel), since the protonation event is prohibited by the lack of histidines. Moreover, the additional arginines and tryptophan confer excessive binding strength to this PBN as discussed above, which further discourages particle disassembly. Accordingly, a delicate balance of forces is required between PBN stabilizing forces (electrostatic, hydrophobic, and hydrogen bonding) and the ultimate pH-sensitive charge destabilizing forces induced by protonation. The additional requirements for maintenance of primary and secondary amphiphilicity to enhance peptide membrane interactions further complicate the task of overall sequence design.

Moreover, the nanoparticle protein corona itself may govern the efficiency of endosomal escape to an extent [135]. A principal mechanism to disassemble particles takes advantage of the physiological process of endosomal acidification by designing naturally pH responsive peptide sequences. As noted above, the inclusion of histidines meets this objective as pH titrates below 5.0 based on the pKa of the imidazole group (~6.0). However, the actual pKa in vivo varies depending on a number of conditions including peptide sequence and neighboring amino acids, ionic environment, corona constituents, polar and nonpolar interactions, and associations with lipid membrane components, such that a range of pKa from 5 to 6.8 is possible [136]. Upon protonation, forces holding the particle together are overcome likely by accumulation of repellant positive charges that lead to disassembly as discussed above. In most reports, this dynamic is confirmed in vitro by treatments with bafilomycin that prevent endosomal acidification, which would inhibit endosomal release since the permeabilizing peptide is no longer free to engage the endosomal membrane [51,52]. For this mechanism, Hou et al. have demonstrated that protonation of only two histidines per peptide would be required to disrupt the p5RHH peptide sequence [51,52], although for other peptides this would depend on the overall binding energies of the specific complex. Other confirming experiments for pH responsivity include titrating pH in solutions of PBN to demonstrate breakdown of the complexes and the release of nucleotides which can be monitored with dyes that fluoresce only upon combining with free nucleotides, as illustrated by Hou et al. [51,52]. Alternatively, for branched peptide structures held together by cysteine disulfide bonds, a reduction of S-S bonds by glutathione (GSH) to break down the branched complex is possible in a cancer cell environment where GSH is reported to be overexpressed in selected tumor types [137], as demonstrated below in hyperbranched PBN examples.

Third, the endosomal/endolysosomal membrane must become permeable to facilitate intact nucleotide escape into the cytoplasm. As mentioned for PBN, the liberated peptide moiety is the critical actor in one of several ways. Upon interaction between phospholipid head groups and basic peptide residues, secondary structures such as alpha helices can create facial amphiphilicity, which in addition to segmented hydrophilic and hydrophobic amphiphilic domains (primary amphiphilicity) can establish the conditions for stable membrane insertion and pore formation [29,47,132,138,139,140,141,142]. Alternatively, the free peptides can act as cationic surfactant detergents or dispersants to disrupt bilayer membrane order and membrane curvature that permits egress of free nucleotide [143]. For example, melittin exerts a prominent membrane thinning and disordering effect on lipid tails that alters macroscopic structure to either form small pores or create leaks that enhance transmembrane diffusion [86,144]. In the case of melittin, Ladokhin has reported that the peptide-to-lipid ratio sufficient to disrupt synthetic lipid vesicles is ~1:50, indicative of its potency as a membranolytic agent [145,146]. Here, Ladokhin also makes the point that lipid membrane composition can dramatically affect these ratios that induce membrane leaks. By contrast, Hou reported that P5RHH, the highly modified version of melittin that readily forms PBN with siRNA, exhibits an LC50 for red blood cell hemolysis that is 588× greater than that for native melittin (see below) [51]. Accordingly, only when the p5RHH PBN is highly concentrated in small endosomal compartments and disassembled from the PBN would it exert a membrane-destabilizing effect to release the nucleotide cargo as observed upon protonation of its histidine residues. In this case, the peptide:lipid ratios required for membrane disruption would be orders of magnitude greater for p5RHH than for melittin. For the interested reader, a more comprehensive discussion of the thermodynamics and physical mechanisms of lipid membrane disruption by surfactants and amphipathic peptides can be found in a review article by Heerklotz [147].

A third opportunity for membrane disruption is referred to as the “proton sponge “effect where the histidines buffer sufficient protons that are accompanied by counterion ingress to change osmolarity enough that water equilibration and swelling mechanically disrupt the endosomal membrane [148,149]. For this mechanism, in some cases protonation of as many as 10 histidines per peptide might be required to osmotically destabilize membranes [150]. This osmotic mechanism is felt to be responsible in part for the action of chloroquine and other endosomolytic agents on endosomal permeabilization and nucleotide escape [133,151].

Fourth, the complexing peptide “excipient, “once disassembled from a PBN, must not itself exert any whole cell cytotoxicity. Apparently, this was a problem for the Arrowhead formulation of masked melittin, when upon unmasking and wide distribution the liberated native melittin presumably engendered significant cytotoxicity to normal tissues resulting in abandonment of the structure. In contrast, the work of Pan, Hou, and colleagues created the peptide sequence p5RHH, a highly modified version of melittin, which exhibits no observable toxicity in vivo in circulation but is critical for rapid and extensive endosomal escape (see below). The LC50 for melittin (0.5–2 mM) vs. free p5RHH (~300 mM) provides a significant margin for safety in circulation (i.e., nearly three orders of magnitude), in addition to the inability of the p5RHH complex to disassemble in circulation at neutral pH, which further prevents dissociated free p5RHH in circulation [51,52]. Unfortunately, most reports of other PBN structures do not quantify LC50 values for the peptide component for comparison, except as noted below.

## 6. PBN Applications In Vivo

### 6.1. Linear Cationic Peptide Self-Assembled Nanoparticles

#### 6.1.1. Transportan/PepFect/NickFect

This family of short peptides contains both linear and branched members and has been developed and reported extensively by the Langel group [28,70]. The original peptide, galparan, was created as a chimeric fusion of a fragment of the human neuropeptide, galanin and a peptide component of wasp venom, mastoparan: GWTLNSAGYLLG-P-INLKALAALAKKIL. Subsequent replacement of the central proline (P) with lysine (K) gave the peptide transportan (“TP “: GWTLNSAGYLLG-K*-INLKALAALAKKIL), which was then shortened by truncation of six N-terminal residues to yield transportan 10 (“TP10 “: AGYLLG-K*-INLKALAALAKKIL). Other modifications then produced the linear PepFect series and NickFect families. Unmodified TP itself does not combine noncovalently with nucleotides to form nanoparticles, so both families have been stearoylated at the N-terminus with the octadecanoic saturated fatty acid (stearate) to provide an extended hydrophobic tail to the amphipathic cationic peptide that will self-assemble into a nanostructure. A comprehensive review by Langel of these myriad structures, their history, putative mechanism of action, and their applications is available to interested readers [70].

Native TP exists as a random coil in solution and assumes a mostly alpha helical secondary structure in lipid membranes, much like other cationic amphipathic peptides. Although TP10 is a ready pore former in lipid membranes, the PepFect constructs PF3 and PF6 require higher concentrations for pore formation. Some stearoylated PepFect analogues (PF14, NF55) have lysines substituted with ornithines that forms a negative nanoparticle complex with splice correcting oligonucleotides at a molar peptide: nucleotide ratio of 5. Histidines have been substituted in some structures to render them pH responsive for enhanced endosomal escape. Membrane permeabilization has been demonstrated in lipid vesicles by dye leakage evaluation. Complexation with nucleotide (plasmid DNA) appears to inhibit membrane interactions. Cell uptake of complexes is described as endocytotic through several standard mechanisms. How these complexes disassemble for cytoplasmic release is not clear. Literally hundreds of PepFect/NickFect analogues exist, with both subtle and major differences in chemistries and behaviors, so it is difficult to generalize even their in vitro fundamental behaviors. Below we summarize recent representative in vivo reports.

In an effort to optimize delivery of plasmid DNA (luciferase) with PF14 (CH3(CH2)16-CONH-AGYLLGKLLOOLA**AA**ALOOLL-NH2; charge = +5), modifications to the structure and complexing ratios of N:P were examined [152]. The substitution of lysine residues (TP10 contains four lysine residues) with ornithine in PF14 (four ornithine residues and one lysine residue) substantially increased the transfection of pDNA and mRNA due to stable complexation with ornithine [153,154]. Additional benefits of ornithine replacement include enhanced resistance against proteases and nucleases [155,156].

**Figure 6:**
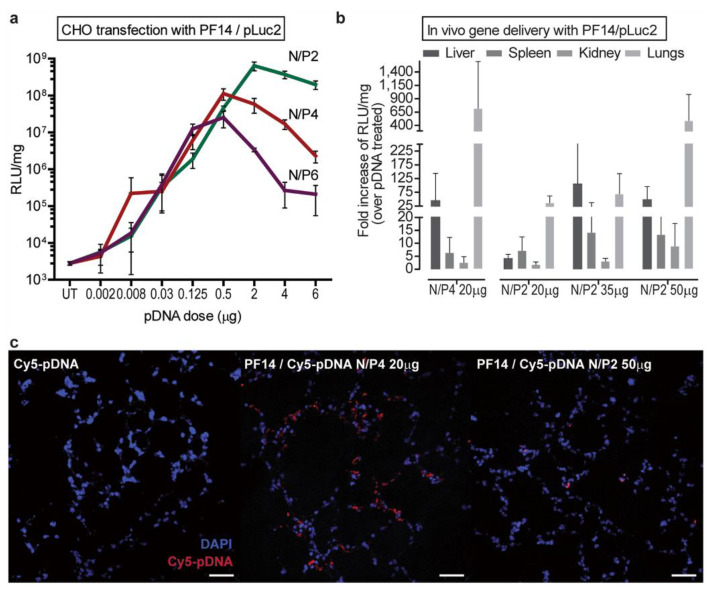
Optimization of plasmid DNA delivery with PepFect14. (**a**) The effect of increasing pLuc dose (up to 6 μg) on transfection efficacy in CHO cells using three different N/P ratios for complexing PF14 with pDNA. (**b**) In vivo reporter gene delivery efficacies of CPP/pLuc at N/P4, compared to N/P2. pDNA doses, formulated with PF14 at indicated N/P, are shown in the X axis. Data are represented as a fold increase of RLU/mg over sham treatment (using the same dose of naked pDNA). (**c**) Accumulation of PF14/pDNA nanoparticles in lung tissue, using Cy5-labeled pDNA. The cryosections of the lungs represent 1 h post-injection. Scale bar 50 μm. From Kurrikov et al. [152].

In vivo evaluations revealed that both P14 and a novel structure, C22-PF14-O, were optimal for pDNA transfection of liver and lung. C22-PF14-O (CH3(CH2)20-CONH-AGYLLGKLLOOLA**OO**ALOOLL-NH2; charge = +7) featured a lengthened N-terminal hydrocarbon chain (C22) and additional + **O**rnithines replacing **A**lanines. For PF14, a reduction in N:P from 4 to 2 and increased dosing enhanced transfection efficiency in lungs (Figure 6). The C22-PF14-O modifications at N:P = 2 enhanced liver transfection. In both cases, stability to proteinase digestion was maximized. Both constructs retained high positive charges after complexation with pDNA (~+30 to +40), and sizes ranged from 100 to 130 nm. Organ toxicity was examined in a subsequent paper [157], and these agents were deemed to be “safe “based on limited histopathological examinations.

In a follow up report, pulmonary inflammation models (LPS lavage and asthma) were developed to test siRNA or plasmid DNA (encoding a small hairpin RNA) against TNF-alpha [157]. Both PF14 and a NickFect agent (NF55: (Stearyl-AGYLLG)δ-OINLKALAALAKAIL-NH_2_) were used to formulate either siRNA and DNA separately in nanoparticle complexes for i.v. therapy. Reductions to variable extents were observed for TNF-alpha mRNA levels (~20–90%) and lung inflammation scores (~0–60%) depending on the disease process and the treatment, with efficacy against asthma models being less evident than for LPS induced inflammation models. Most of the plasmid delivery by PF14 was observed to track to the liver. The authors concluded that these two peptides act as selective “lung targeted “agents, which may be accounted for in part by their large positive charge (~+30).

Other laboratories have utilized PepFects or modified versions of stearoylated transportans for in vivo therapeutic delivery of various cargos. The Bhatia group reported the use of a “tandem peptide “construct containing the myristoylated transportan fused to a PEG spacer with a C-terminal targeting peptide, iRGD (cyclic peptide of CRGDKGPDC), for pancreatic tumor delivery of anti-KRAS (WT) siRNA [158]. After subcutaneous implantation of KPC tumor segments that had been “pre-matured “in mice pancreas, mice were dosed starting on day 1 post-implant for 10 doses (0.5 mg/kg i.v.) over 5 weeks, which reduced tumor size by ~50% and improved survival statistics. In another study by this group, tandem peptides carrying oligonucleotide ligands targeted to selected Toll-like receptors together with tumor homing peptides were injected *intratumorally* to improve responsivity to immune modulators (CTLA4, i.p./wk) against B16F10 melanoma tumors [159]. In murine ovarian cancers, van den Brand et al., deployed PepFect14 carrying a biomarker mRNA (i.e., a fluorophore) by intraperitoneal injection to demonstrate uptake by tumor cells as well as fibroblasts and immune cells, but with no expression of the fluorophore beyond the peritoneal cavity [160].

#### 6.1.2. p5RHH

The 21 amino acid cationic amphipathic peptide “p5RHH “(VLTTGLPALISWIRRRHRRHC) was developed as a biocompatible modification of the natural bee venom peptide melittin by the Wickline group (see Figure 1). Their earlier work with melittin as an anticancer agent employed a microfluidic formulation process with a perfluorocarbon core material (perfluoro-octyl-bromine: PFOB) where melittin was stably inserted into a surfactant lipid monolayer that invested the PFOB core to yield a ~200 nm spherical particle [45,46,49]. Melittin was known to insert into lipid membranes in the traditional manner described for cationic cell penetrating peptides by first interacting with negative lipid head groups electrostatically, assuming an alpha helical secondary structure, and pore forming by oligomerization. This process was enthalpically favorable and exothermic, resulting in a tightly bonded peptide in the PFOB nanoparticle that did not release the melittin in circulation in vivo until encountering cancer tissue environment where it exerted profound local cytotoxicity.

The goal to develop native melittin itself as a peptide-based carrier for nucleotides met with little success in their hands as formulations were not transfective (unpublished data: K.K. Hou et al.). Their next steps sought to reduce the membrane disrupting toxicity of native melittin to design a modified version of melittin as a cargo carrier for stable insertion into cellular or liposomal membranes without causing damage [50]. The truncation of seven N-terminal hydrophobic amino acids reduced red cell membrane disruption by ~300× (LC50: ~150 microM, unpublished observation). A peptide fusion product with a conjugated VCAM-1 (vascular cell adhesion molecule 1)-targeting peptide was created that inserted into lipid membranes stably without disruption or leakage. This peptide, peptide-5 (p5, for the 5th candidate tested), was shown enable post-formulation lipid membrane editing to enhance efficacy of the nanoparticulate cancer drug Doxil^TM^ (i.e., doxorubicin enveloped in a “stealth “liposome) by enabling direct binding to cancer endothelium and uptake via the VCAM-1 targeting peptide fused to the p5 peptide [34,36,50,161]. Unfortunately, p5 also was not transfective for nucleotides.

Additional efforts to further modify p5 at the C-terminus yielded a safe and transfective polyplex with either siRNA or mRNA cargos [51,52,69]. The addition of two histidines and substitution of several arginines produced a 21 amino acid cationic peptide p5RHH that exhibited both primary and secondary amphiphilicity. The p5RHH peptide rapidly condensed siRNA within 40 min by simple mixing (~1:100 ratio for siRNA:peptide; ~1:3000 for mRNA, depending on RNA size) into a ~60 nm polyplex with a charge +12 mv. The LC50 of free p5RHH was ~300 microM, an overall enhancement in safety of ~588× versus free melittin (melittin LC50: 0.51 microM [45]) [51].

The formed polyplex is precoated with an albumin corona that reduces overall charge to –5 mv, improves particle stability, and confers dysopsonization to avoid liver clearance [124,125,126,127,128]. It is resistant to RNAase in circulation and is taken up by macropinocytosis. The structure exhibits a pH dependence such that when captured in an endosome under pH < 4.5, two histidine residues (pKa ~6.2) are protonated on the peptide carrier, which then disassembles. The free peptide now in high concentration disrupts the endosomal membrane, which rapidly and extensively releases RNA cargos into cytoplasm. The system avoids uptake by the RES (liver and spleen), due to small size and corona precoating, and does not cross normal vascular barriers yet can passively permeate inflammatory pathologies and leaky vasculature in vivo. Renal clearance appears to predominate [66,115,129,162], although the exact mechanism by which this occurs is not certain. However, various pathways have been described for nanoparticles larger than the glomerular filtration barrier such as capillary permeation through transcytosis and proximal tubule uptake, active podocyte uptake and transport of trapped albumin-coated particles, or polyplex breakdown into smaller filterable peptide-RNA units or component separation at the glomerulus [163]. Preliminary estimates of circulation reveal T_1/2_ ≈ 43 min (1-compartment model) [164].

Endosomal membrane permeabilization is likely a consequence of the detergent (i.e., cationic surfactant) action of p5RHH as described above, although pore formation cannot be ruled out. Proton sponge release mechanisms cannot be excluded, but the presence of only two histidines per peptide likely militates against this. Extensive endosomal escape is notable for p5RHH nanostructures as chloroquine yields no additive effect to siRNA knockdown in vitro [51,52]. Moreover, once released, the free peptide has been shown to be nontoxic to cells in the doses used as it is rapidly diluted in the cell cytoplasm.

**Figure 7:**
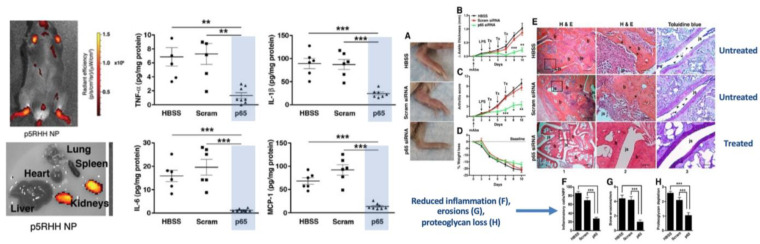
Systemic Extrahepatic Delivery of p5RHH-siRNA. Polyarticular arthritis induced by anti-collagen antibodies and LPS in mice treated with anti-NF-kB (p65) siRNA-p5RHH PBN. Note delivery to inflamed paws and renal clearance with no appreciable deposition in liver or spleen 24 h after i.v. injection (**left panels**). Broad and potent suppression of inflammatory cytokines in p65 treated subjects (shaded in blue) were observed (**middle panels**), where triangles, circles, and squares represent buffer (HBSS), scrambled siRNA (Scram), and p65 siRNA (p65) treatments respectively. Beneficial reductions of inflammation and joint destruction 11 days after three serial i.v. doses (**right panels**). (**A**) shows the reduction of paw inflammation after p65 treatment. (**B**,**C**) show reduction of ankle thickness and arthritis score, respectively; and (**D**), no effect of treatment on weight. (**E**) shows joint histology indicating reduction of inflammation (scale bars: 400 μm (left), 100 μm (middle), 50 μm (right). (**F**–**H**) show the effect of p65 siRNA treatment on inflammatory cells, bone erosions and proteoglycan depletion. Stars (*) indicate significant differences in all panels. Adapted from Zhou et al. [129].

Numerous in vivo studies of efficacy in small animal models using both siRNA and mRNA for systemic extrahepatic delivery have been reported by independent academic laboratories collaborating with this group. In collagen antibody-induced arthritis, canonical Nuclear Factor Kappa-light-chain-enhancer of activated B cells (NF-kB) was targeted with p65 siRNA polyplexes (Figure 7) [129]. After only three sequential doses of i.v. (0.5 mg/kg siRNA), broad spectrum cytokine (IL-6, IL-1beta, TNF-alpha, MCP-1) suppression was observed in joint synovium resulting in reduced inflammation, paw swelling, and bony erosions and restored joint glycosaminoglycans. Knockdown of p65 was selective as other members of the NF-kB family were unaffected. Moreover, there were no organ/tissue/blood toxicities, no suppression of p65 in other organs, no induction of adaptive immunity (IgG/IgM levels to polyplex or to the free peptide), no effects on innate immune responsivity, and no complement activation. Clearance was renal, and minimal accumulation in liver or spleen was observed. Several follow-up studies in post-injury mouse knee osteoarthritis (OA) employed single local injections of p65, mixed p65/p100, or other targets of siRNA polyplexes to show similar control of inflammation and joint destruction as well as sustained pain relief [162,164,165].

Other recent in vivo studies of cancer in mouse models have been completed against various tumor driver molecular targets. In pancreatic cancer, wild type KRAS was targeted with siRNA(unmodified)-p5RHH polyplexes administered systemically in serial doses to subcutaneous KPC-1 PDAC xenografts. KRAS siRNA uptake was observed in >85% of cancer cells isolated from the tumor mass, and tumor growth was suppressed by ~80% [166]. Deep penetration into the tumor mass was observed in spontaneous genetically engineered KPPC PDAC tumors with dense stroma, but not into normal pancreatic tissues or other organs. In Adult T-cell Leukemia/Lymphoma models driven by the HTLV/Tat oncogene and constitutively expressed NF-kB, serial p65 siRNA systemic dosing fully suppressed tumor growth in the rapid growing tumor cohorts [167]. In ovarian and uterine cancer models, siRNA targeting AXL, a driver of tumor cell motility and metastasis, was administered intraperitoneally to suppress tumor nodule number and mass [168]. In a mouse metastatic melanoma model, p65 siRNA polyplexes administered systemically in a single dose suppressed metastatic tumor expansion in the lungs by 80% [169].

Lung and sarcoma tumor xenografts were shown to be susceptible to siRNA polyplexes against factors that control tumor angiogenesis (ETV-2 and MYCT1) and the immunosuppressive tumor microenvironment [170,171]. In addition to marked suppression of tumor growth, tumor neoangiogenesis was inhibited, indicating the potential for development of a new class of antiangiogenic therapies employing PBNs targeted to upregulated tumor vascular growth factors. Additionally, enhanced responsivity to immune modulation was achieved with combinations of p5RHH-siRNA polyplexes, check point inhibitors (anti-PD1), and conventional antiangiogenic agents to either completely suppress or fully regress tumor masses in the majority of treated animals [171].

Other broad-ranging in vivo studies using siRNA polyplexes have included metabolic syndrome, necrotizing enterocolitis (NEC), and atherosclerosis. In fat-fed mice that develop obesity, targeting ASXL2 that drives white fat inflammation, macrophages were converted from inflammatory to resolving phenotypes; adipose inflammation was reduced, and weight gain was inhibited [172]. In mouse models of NEC, a highly inflammatory process of progressive bowel necrosis in premature infants that have no specific medical therapy, NF-kB canonical p65 siRNA polyplexes delivered systemically in a single dose markedly suppressed bowel inflammation and cellular infiltrates, preserved bowel architecture, and prolonged survival [173]. In atherosclerotic fat-fed ApoE null mice, JNK2 targeting with siRNA dosed 2×/wk for 3.5 weeks (0.5 mg/kg i.v.) resulted in reduced: (1) macrophage NF-kB and STAT-3 expression, (2) macrophage content, (3) plaque necrotic core, (4) endothelial barrier disruption, (5) thrombotic risk, and (6) aortic plaque coverage [114].

Additional studies of mRNA delivery with p5RHH polyplexes have been reported. Ex vivo studies in explanted cartilage from patients with OA utilized mRNA to enhance production of WNT16 (~1100 nt), which itself antagonizes the WNT3/beta-catenin inflammatory pathway that has been implicated in the development of OA [67]. In this study, hyaluronic acid (HA) was used as the nanoparticle coating to enhance uptake in the joint as the cognate receptor for HA is CD-44, which is prominently expressed in joint tissues and chondrocytes. At an N:P ratio of 3500:1, particle sizes were ~65 nm and charge ~–30 mv. Deep cartilage penetration, sustained production of WNT16, inhibition of WNT3 and beta catenin, reduction of cartilage apoptosis, and restoration of lubricin levels (an essential joint lubricant) were observed.

A recent study of systemic delivery of p27^Kip1^ mRNA in a mouse model of vascular endothelial damage and restenosis sought to attenuate vascular smooth muscle cell participation in intimal-medial hyperplasia [66]. p27 is a cyclin-dependent kinase inhibitor that regulates cell cycling at the G1 stage. In this study, the p27 mRNA was fused with an endothelial specific miR-126 target sequence to allow overexpression of p27 in smooth muscle cells but suppression in endothelium by miR-126 recognition and clearing of the p27mRNA sequence only in endothelial cells. This strategy would therefore permit endothelial recoverage of the vascular surfaces that are damaged during angioplasty to reduce prothrombotic exposure. Clotting risk is a significant problem for antiproliferative drug eluting stents deployed during angioplasty due to the marked slowing of endothelial recovery that necessitates a year or more of systemic anticoagulation with its attendant bleeding risk. Systemic administration of mRNA over several weeks (five doses @~0.25 mg/kg over 2 wks) resulted in selective delivery to the lesion areas and overexpression of p27 only in the vascular lesions themselves. Endothelium repopulated the damaged vascular surfaces, and restenosis was markedly attenuated. These peptide polyplexes of p5RHH-RNA are being developed commercially by Altamira Therapeutics, Inc., (Dover, DE, USA).

#### 6.1.3. GALA/KALA/RALA

The cationic 30-mer KALA peptide (WEAKLAKALAKALAKHLAKALAKALKACEA) designed by the Szoka group is amphipathic and exists in alpha-helical configuration at neutral pH [174]. The negatively charged precursor peptide, GALA, was modified by substituting a number of positively charged lysines for glutamic acids to render it cationic and facially amphiphilic with lysines dominating the hydrophilic side opposite the hydrophobic leucine residues. Interestingly, as pH drops to ≤4.5, the glutamic acid side chains are neutralized, and the histidine is protonated, resulting in an increase in net positive charge that was observed to reduces its helicity from 45% to 24%. However, KALA can bind oligonucleotides and plasmid DNA at an optimal charge ratio of 10/1 (+peptide/-nucleotide), which renders it transfective. Either as a free peptide or when complexed to nucleotides, it can interact with and disrupt membranes depending on pH and composition, presumably by pore formation.

KALA was not destined to become a utile PBN former perhaps due to its nonselective membrane disrupting activity at neutral pH. With that in mind, McCarthy et al. modified the KALA peptide by substituting arginines for lysines in the RALA motif (WEARLARALARALARHLARALARALRACEA), which retains the cationic charge and facial amphiphilicity in alpha helical form [175]. The arginine residues were surmised to enhance DNA binding and transfection by electrostatic interactions. Although the mechanism of endosomal particle disassembly was not evaluated, a pH dependence for in vitro lipidic membrane disrupting activity for the RALA/DNA complex was noted at pH = 5.5. The pH responsiveness within acidifying endosomal structures would be retained for the single histidine residue by protonation (pKa of imidazole group ~6) and perhaps for the glutamic acid (pKa of second carboxyl group = 4.15) residues depending on the ultimate acidity of the endosomal environment.

When combined with plasmid DNA at an N:P ratio of 10:1, RALA self assembles a 51 nm nanoparticle with a charge of +29 mV and polydispersity index (PDI) of 0.35. Transfection efficiency in vitro ranged from 25 to 60% depending on cell type. Although the pH dependence of endosomal escape was not evaluated experimentally in the study with the use of bafilomycin to inhibit endosomal acidification, chloroquine elicited no additional endosomal release at the N:P ratio of 10:1. Biodistribution studies after systemic administration of luciferase RALA/DNA in mice indicated primarily lung and liver delivery of plasmids based on ex vivo organ incubation with luciferin and bioluminescence expression at days 2 and 7. Later attempts to reduce the size of the sequence through truncations/substitutions of selected residues were not successful [176].

Two in vivo studies featuring systemic administration of RALA-nucleotide complexes have been carried out in cancer models with inducible nitric oxide synthase (iNOS) as the therapeutic entity [177,178]. For metastatic breast cancer evaluation, RALA complexed with plasmids encoding iNOS was administered i.v. (five doses, 2×/wk) to mice that had been inoculated intracardiac 48 h prior with a breast tumor cell line. Mice were euthanized when 20% body weight loss had occurred. Median survival time was increased by 27% compared with controls. The combination treatment of docetaxel and RALA/iNOS was no more effective than docetaxel alone for medium survival time, although tumor bioluminescence appeared more delayed in the combination treated cohort. For metastatic prostate cancer evaluation, RALA complexed with similar plasmid encoding iNOS was delivered i.v. (five doses, 2×/wk) to mice that had been inoculated intracardiac 48 h prior with a prostate tumor cell line. Median survival time was extended by 56% in the treated mice versus RALA only controls.

Another in vivo study compared the local tumor injection of siRNA and plasmid DNA targeting the FK506-binding protein such as the –FKBPL gene (pFKBPL). FKBPL is a member of the immunophilin protein family that may inhibit angiogenesis and tumor growth by CD44 receptor dependent mechanisms [179]. RALA DNA complexes were prepared as above and administered *intratumorally* (10 mg, 2×/wk) to mice with established intradermal breast tumor xenografts. The plasmid DNA treatment resulted in upregulated production of FKBPL in the tumor, reduced vasculature, reduced tumor sizes, and prolonged survival times. The anti-FKBPL siRNA (2 mg, 2×/wk) did not affect tumor size but did appear to increase tumor angiogenesis. Other studies of local *intradermal* injections for RALA vaccine formulations also have been reported [180,181]. RALA peptide-RNA polyplexes are being developed commercially by pHion Therapeutics, Inc., (Belfast, Northern Ireland).

#### 6.1.4. WRAP

Boisguerin, Deshayes, and colleagues recently reported the design of a family of short cationic CPP called “WRAP “that are composed of trytophan (W) and arginine (R) amphipathic (A) peptides (P), which rapidly form ~100 nm PBN upon mixing with nucleotides and incubating for 30 min [84]. The authors reasoned that such structures featuring tryptophan with its aromatic, hydrophobic indole ring that might potentially “pi bond “with other nucleotide ring structures while also contributing hydrophobic facial amphiphilicity, along with leucines, when in alpha helical secondary structures. Arginines would alternatively serve as hydrophilic components of amphiphilicity as well as hydrogen bonding partners with various nucleotide constituents. Based on a series of in vitro measurements of physical characteristics and activities, WRAP1 (LLWRLWRLLWRLWRLL: 16-mer, 4 W, +5 charge) and WRAP5 (LLRLLRWWWRLLRLL: 16-mer, 3 W, +5 charge) were specified as candidate agents for formulation of PBN.

When mixed with siRNA, circular dichroism analysis demonstrated that both peptides existed in alpha helical form. The molar ratios of WRAP:siRNA resulting in stable PBN ranged from 20 to 40 in vitro, yielding sizes by DLS (intensity) of 73 and 80 nm, zeta potentials of +42 and +29, and PDI of 0.38 and 0.29, respectively, for WRAP 1 and WRAP5 (measured at molar ratio = 20). Interestingly, the number distributions by DLS suggested that these average measurements represented less than half of the particles in the measurement cuvette. From the images provided, it appears that these 100 nm structures are strings of smaller particles that have aggregated from more globular smaller particles. The DLS measured sizes did not change over 72 h at 4 °C. Subsequent studies were done with a molar WRAP:siRNA ratio of 20:1, which represents an approximate charge ratio of 1:4.

Knockdown was tested in vitro against a variety of cell types stably expressing luciferase. At an anti-luciferase siRNA concentration of 20 nM, relative knockdown of ~50–80% for fluorescence activity across all lines or CDK4 (cyclin dependent kinase-4) in U87 human glioblastoma cancer cells was observed after 36 h. In cell internalization experiments in vivo, fluorescence was observed in a punctate pattern after incubation for 1 h, suggesting compartmentalization under these conditions. Interestingly, 50% of maximum uptake for WRAP5 occurred after only 3 min, and maximum uptake was achieved by 15 min. Knockdown of luciferase activity and CDK4 signal exceeded 40% after only a 5 min exposure to PBN when measured 36 h later. Maximal knockdown was observed after only 60 min exposures.

Of note, a later publication failed to identify any of the usual endocytic compartments as colocalizing with the PBN, and incubation in cold or energy depleted conditions did not prevent uptake, whereupon the authors concluded that active endocytosis was unlikely to be the main uptake mechanism [182]. They did report some colocalization with transferrin however, suggesting at least a modicum of endocytosis. Additionally noted was the substantial membrane disruption of large unilamellar lipidic vesicles by both free (2.5 mM) and siRNA-complexed (2.5 mM) WRAP peptides within ~100 s. Data on red blood cell lysis were not provided. EM images confirmed a degree of sorting into membrane-confined compartments, but these were not colocalized with early or late endosomal or lysosomal markers.

In an in vivo experiment to define the ability of WRAP-siRNA to achieve target knock down, an orthotopic brain tumor model was prepared with stereotactic implantation of U87 glioblastoma cells expressing luciferase [183]. WRAP5 loaded with anti-luciferase siRNA (20:1 ratio; 20 mg) was *injected directly* into the tumors under stereotactic guidance. A reduction of luciferin fluorescence of ~70% was observed after 24 h, but tumor growth was not affected. Tumor cells, as well as surrounding macrophage and matrix cells, were noted to take up the PBN.

## 7. Branched Peptide Self-Assembled Nanoparticles

### 7.1. “HK” Branched Polymers

Mixon and colleagues have been working for some time on refining synthetic branched polyplexes containing various sequences of histidine and lysine, called “HK peptides “(see Figure 3) [184,185,186]. These and other efforts date back to initial work by Midoux and Monsigny [187] and the Bechinger group describing the role of histidine in polylysine complexes for the packaging of DNA, endosomal escape, and transfection [188,189]. These peptides form both ionic and nonionic bonds with nucleotides to condense them into polyplex format. In a recent representative update, they assembled and tested various sequences for plasmid DNA complexation and transfection, as well as tumor selective targeting aspects [186].

The peptide polyplexes tested comprised a tri-lysine core with four terminal branches of linear lysine-histidine peptides covalently coupled in peptide bonds. The linear branches were of differing lengths from 12–20 amino acid residues, but each branch was of the same length in each polyplex structure. The repeating motif in the branches was “H2K “(e.g., H2K4b-14: [KHHKHHKHHKHHHK]_4_LYS, a 14-mer peptide with 5K and 9H). Luciferase plasmids were complexed with the peptides in ratios of 1:2 and 4:1, mixed by pipetting and incubated for 45 min. Transfection of tumor xenografts in vivo after i.v. injection revealed that the H2K4b-14 polyplex (with 414-mers) formulated at a ratio of 1 peptide:2 plasmid DNA was most successful. Interestingly, this complex was negatively charged (–18 mV; d = 150 nm) zeta potential) as compared to the 4:1 ratio polyplex (+25 mV; d = 98 nm) that was less transfective. Tumor activity of the luciferase was >5x that observed in lung, and even less signal was observed in liver and spleen.

The authors also evaluated the incorporation of other endosomal disrupting and endothelial targeting peptides into the polyplex. Adding a linear 33-mer (H3K-33: 11K, 22H) endosomal disrupting peptide to the mix along with a branched cRGD targeting agent against tumor αvβ3/β5 integrins (cRGD-PEG-H3K4b) led to improved transfection in vivo (tumor/lung ratio ~13). A role for NRP-1 (neuropilin-1) in mediating in vivo tumor uptake of the polyplex was demonstrated by preblocking with an antibody against NRP-1, which reduced tumor luciferase activity by 98%. NRP-1 is a receptor for CendR motif peptides that may be created by protease processing of the branched peptides resulting in exposure of a K/RXXK/R segment that binds to NRP-1 for transport into the cell [97]. The RGD peptide first binds to tumor endothelial αvβ3/β5 integrins where the cleavage and exposure of the CendR motif (KXXK) occurs to create the NRP-1 binding segment.

Although these specific structures are not yet evaluated for therapeutic efficacy, the authors have demonstrated a theoretical benefit versus a linear version of HK peptides that has been evaluated in vivo, 20-mer H2K. This peptide was also transfective but seemingly less stable to trypsin digestion than its quad-branched relative, H2K4b-20 with the same amino acid sequence in the branches, which motivated the exploration of shorter branched structures [184]. It is also notable that these branched polyplexes contain no hydrophobic segments that are thought to potentiate membrane interactions and endosomal escape, although the addition of the disruptive and targeting peptides likely served this purpose. Versions of branched HK polypeptide RNA structures are being developed commercially by Sirnaomics, Inc. (Germantown, MD, USA).

### 7.2. Branched Amphiphilic Peptide Capsules (BAPCs)

This interesting class of peptides developed by the Tomich group comprises self-assembling liposome-like structures with a water-filled cavity surrounded by an amphiphilic branched dipeptide bilayer [190,191]. The peptide bilayers form after incubation for 2 h of two branched peptides (1:1 ratio) containing 15 and 23 amino acid residues. Five positive charges (all lysines) are positioned on one side of the sequence with the fifth N-terminal lysine that forks off two equivalent mostly hydrophobic segments: FLIVI and FLIVIGSII for the 15-mer (bis(Ac-FLIVI)-K-K4) and 23-mer (bis(Ac-FLIVIGSII)-K-K4), respectively. The rationale for the incorporation of two sized peptides was to accommodate membrane strain due to curvature of the formed vesicle. The hydrophobic segments are poorly soluble in water and seek to form a 4 nm thick bilayer that envelopes the water filled core of the unilamellar structure. The initial phases of assembly involve smaller 20 nm capsules that progressively fuse over time. The fusion process can be stopped by cooling, which seems to stabilize the particles that then are no longer susceptible to further fusion even after rewarming. The resulting structure is a 20–30 nm vesicle that is held together in part by hydrogen bonding with the hydrophilic polylysine segments arranged on the faces toward the aqueous phases (charge: ~+55 mV).

**Figure 8:**
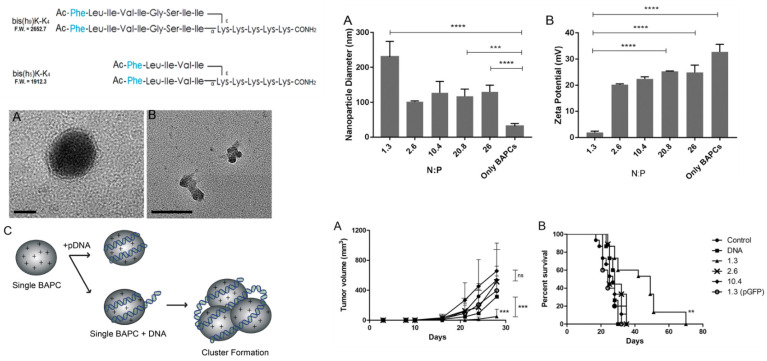
Branched amphipathic peptide capsule (BAPC) forming nanoparticles. (**Left**)*:* BAPC sequences and TEM images of the BACP:DNA nanoparticles at N:P = 20.8. (**A**) Single BAPCs interacting with pDNA. Scale bar = 10 nm. (**B**) Cluster of BAPCs interacting with DNA. Scale bar = 100 nm. (**C**) Schematic representation of potential BAPC-DNA interactions. (**Right top**)*:* Dynamic light scattering (DLS) and zeta potential data for different BAPCs-DNA formulations. (**A**) Size (z-average) and (**B**) zeta potential. (**Right bottom**)*:* Antitumor effect and survival curves of mice immunized with BAPCs-DNA nanoparticles at different N:P ratios. C57BL/6mice were immunized i.m. with plasmid DNA (40 μg) 3 days after injection of tumor cells (TC-1) complexed with or without BAPCs at 1.3, 2.6, and 10.4 charge ratios (N:P). The sham-treated group was inoculated with PBS based on the same inoculation regimen. The 1.3 (pGFP) group received 40 μg of pGFP plasmid, used as a negative control for the vaccine. (**A**) Mean values of tumor size (mm^3^) progression +SD values until day 30. (**B**) Survival rates within 70 days after the TC-1 injection. Stars (*) indicate significant differences and “ns “is nonsignificant. Adapted from Avila et al. [192] and Barros et al. [193].

In vivo studies of plasmid DNA transfection for tumor growth suppression were carried out by mixing pDNA with the cationic BAPCs to form 50–100 nm individual structures by electrostatic interactions at the vesicle surface (Figure 8) [192]. Clusters of 100–250 nm also form that appear to be covered with DNA. For GFP plasmids (4.7 kb) testing in vitro at an N:P ratio of 20.8 and charge of ~25 mv, 50% transfection was observed in vitro by 6 h. For in vivo testing against tumor growth, a plasmid-based DNA vaccine was prepared with a human papilloma virus oncoprotein construct (5.6 kb) at various N:P ratios. Only mice immunized with pgDE7-coated BAPCs at N:P of 1.3 delivered i.m. demonstrated tumor growth suppression for up to one month and prolonged survival times. Interestingly, the charge for this species was 2 mv, much reduced compared to the other more heavily peptide loaded species. Features such as the relative neutrality of the particles that might have prevented lung accumulation, cytotoxicity, and reduced plasma protein adsorption and aggregation were suggested as factors accounting for the response. No elevations in markers of liver toxicity were observed.

A follow up study on the complexation of mRNA (GFP) to BAPCs revealed biodistribution primarily to lung and liver early but clearance by 24 h in normal mice [194]. These particles were 50–350 nm in diameter with charge of ~36 mv. Expression of cytokines IL-6, TNF-alpha, and IFN-gamma was elevated at 1 and 3 h after administration. PK and transfection efficiency (GFP expression) were not reported.

### 7.3. B-mR9

**Figure 9:**
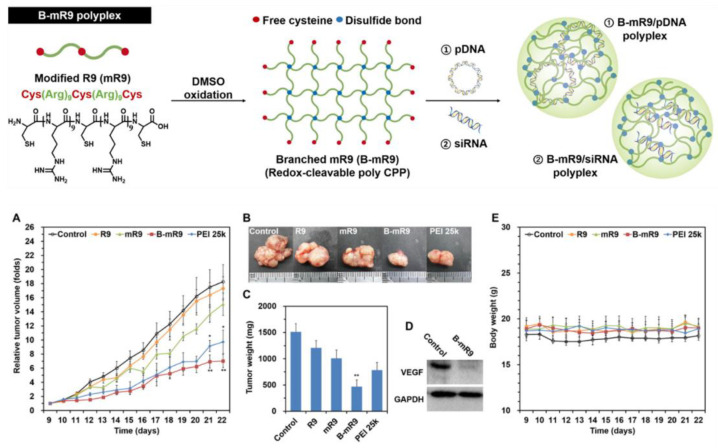
Branched-modified R9 (B-mR9) cell-penetrating peptide (CPP) and nucleotide polyplexes. (**Top panel**)*:* Positively charged B-mR9 combine with negatively charged nucleotides through electrostatic interactions. The cysteines in branched structures of B-mR9 can be cleaved by cellular reducing agents to release nucleotide cargos. (**Bottom panel**)*:* (**A**) Tumor responses (fold changes) after intravenous administration of control, R9, mR9, B-mR9, and PEI 25 k with 20 μg of siVEGF. (**B**) Representative tumor images, (**C**) Tumor weights measured on 22nd day, (**D**) Western blot of tumor VEGF, (**E**) Body weights. Stars (*) indicate significant differences. Adapted from Yoo et al. [195].

These peptides combine a branched polyarginine structure and reducible S-S disulfide bonds with the use of interposed cysteines linkers (mR9: Cys-R9-Cys-R9-Cys) (Figure 9, top panel) [195]. Incubation overnight forms a multi-branched polymerized gel (B-mR9) that can be purified and lyophilized to a molecular weight of 138,889 g/mole. Plasmid DNA polyplexes are then formed by incubation with DNA at various N:P ratios for 30 min. Under reducing conditions (incubation with dithiothreitol), no condensation of DNA was observed as the branching structure was prevented. The average size, charge, and PDI were quoted as ~88 nm, +20 mv, and 0.26, respectively, for DNA complexes. For in vitro testing, an N:P ratio of 15 yielded transfection efficiencies of 30–50% depending on cell type and conditions. Cell viability exceeded 80%, and no red cell hemolysis was observed for the complexes.

In vitro testing was conducted for siRNA polyplexes to suppress VEGF production at an N:P ratio of 10. Size and charge for these complexes were 129 nm and +23 mv, respectively, which were stable in albumin solutions. VEGF knockdown ranged from 30 to 80% depending on cell type but fell by ~50% when cellular GSH production and disulfide reduction was inhibited by pretreatment with buthionine sulfoximine. Cell uptake occurred by energy requiring macropinocytotic and caveolar endocytotic mechanisms. Substantial endosomal escape was documented by 3 h after incubation by conversion from punctate to diffuse cytoplasmic fluorescent siRNA signal intensity.

Efficacy in vivo was tested in an NCI-H460 (non-small cell lung cancer) xenograft murine model (Figure 9, bottom panel), which was the cell type manifesting the highest VEGF knockdown in vitro (~80%). Intravenous dosing of 20 micrograms siRNA 3×/wk was administered over 3 weeks, although it is not clear from the description when the treatments were started. The relative tumor growth rate over the final 2 weeks for the B-mR9 treated mice was ~40% of that for the untreated control group. Tumor VEGF was reduced by 2.3-fold. Body weight remained unchanged in both treated and control groups.

In a follow up study of methotrexate complexed to B-mR9 for tumor inhibition in the same xenografts [196], a coating of hyaluronic acid was added to the polyplexes to engage tumor CD-44 receptors. Again, tumor volumes were reduced after 3 weeks, and body weights were unchanged versus a minor effect on growth for free methotrexate.

### 7.4. Peptide Spiders

In this work, the Bhattia group takes advantage of their prior work with the synthetic antimicrobial peptide, transportan, to create a octad-branched peptide bearing structure grafted onto a an eight-armed PEG molecule [197]. Transportan was developed previously by Langel and colleagues as a conjugated cell penetrating peptide that was derived by fusing natural peptides as described above for PepFect. Transportan analogues also are known to be effective PBN formers. For this spider structure, a transportan peptide and an integrin targeting peptide (iRGD) were coupled to the PEG octad backbone in stochiometric ratios to enable both functionalities in controllable quantities. The resulting “peptide spider “conjugates were mixed with siRNA at peptide:RNA ratios from 0.25 to 8 (N:P = 0.156 to 5) resulting in best performing ratio of 4:1 for in vitro knockdown experiments. The size was in the 50–100 nm range depending on conditions, and the charge was near neutral.

Testing in vivo with the 4:1 ratio material was conducted for knockdown of a transcription factor expressed in a breast tumor xenograft, ID4. Three doses of 1.4 mg/kg siRNA were administered i.v. at 2-day intervals prior to euthanasia, which achieved 60% ID4 downregulation. Interestingly, pharmacokinetic evaluation revealed an extremely rapid clearance half-life of 2.1 min presumably calculated by 1-compartment modeling since most of the material was removed from circulation before a distribution phase could be registered. The agent appeared to be widely distributed to lung, liver, spleen, and tumor tissues without obvious organ toxicities by H&E staining. No tumor growth inhibition studies were reported.

## 8. Conclusions

Although there is no PBN approved for clinical use, a clear medical need exists for systemic delivery of nucleotides for therapy at sites beyond the liver. Because most large biotech and pharmaceutical companies are now focused on mRNA vaccines utilizing ionizable lipid nanoparticle constructs for delivery, interest in new methods of delivery such as PBN are yet to receive the attention they need to rapidly advance early development and clinical testing programs. Other challenges to future product development include stressed commercial production capacity for RNA and peptides, robust formulation processes for manufacturing scale up, design of non-freezing storage methods that maintain stability, and availability of nonhuman primate disease models for toxicity and efficacy testing, among others. Whether molecular targeting will be useful to further enhance their tissue and cell selectivity is conjectural at this point, but it always seems to arise as a question in discussions of the technology. For the moment, the EPR effect appears to satisfy permeation requirements, given that there are no robust and universal targeting approaches for tissues besides the liver. Nevertheless, advocates for this approach tout the flexibility, simplicity, and cost-effectiveness of these self-assembling structures as the next generation of RNA and DNA delivery agents.

Herein, we have reviewed a selection of promising recent reports on PBN that appear to meet the objectives of extrahepatic delivery. In view of the growing interest in the field of RNA therapeutics beyond vaccines, we anticipate that some of these agents and other new ones will emerge in full development and clinical testing programs over the next few years. In particular, the applications of PBN mRNA promise to enable engagement of the missing factor in the pharmacopeia equation, the overexpression of critical proteins that have been *downregulated* in disease processes. Taken together with the conjunctive approaches to suppress proteins that are mutated or overexpressed with the use of small molecules and/or RNA-based inhibitors (siRNA, miRNA, ASO, etc.), fine tuning of adaptive personalized therapeutics may become a reality.

## Figures and Tables

**Table 1:** Selected Peptides Forming PBN.

Linear Peptides	Basic Sequence	Charge
Transportan	GWTLNSAGYLLGKINLKALAALAKKIL	4
Transportan 10	AGYLLGKINLKALAALAKKIL	4
PepFect14	CH3(CH2)16-CONH-AGYLLGKLLOOLAAAALOOLL-NH2	5
C22-PepFect14-O	CH3(CH2)20-CONH-AGYLLGKLLOOLAOOALOOLL-NH2	7
NickFect 55	Stearyl-AGYLLG)δ-OINLKALAALAKAIL-NH2	3
p5RHH	VLTTGLPALISWIRRRHRRHC	5
KALA	WEARLARALARALARHLARALARALRACEA	6
WRAP1	LLWRLWRLLWRLWRLL	5
WRAP5	LLRLLRWWWRLLRLL	5
Branched Peptides		
H2K4b-14	[KHHKHHKHHKHHHK]_4_LYS	56
BAPC	(Ac-FLIVI)2-K-K4-CO-NH2; (Ac-FLIVIGSII)2-K-K4--CO-NH2	5;5
B-mR9	mR9: Cys-R9-Cys-R9-Cys	18
“Spiders“	[GWTLNSAGYLLGKINLKALAALAKKILC]6-PEG	24
